# Whole‐brain glucose metabolic pattern differentiates minimally conscious state from unresponsive wakefulness syndrome

**DOI:** 10.1111/cns.14787

**Published:** 2024-06-18

**Authors:** Kun Guo, Qi Zhang, Zhiyong Quan, Yirong Wang, Taoqi Ma, Jiehui Jiang, Fei Kang, Jing Wang

**Affiliations:** ^1^ Department of Nuclear Medicine, Xijing Hospital Fourth Military Medical University Xi'an China; ^2^ School of Communication & Information Engineering Shanghai University Shanghai China; ^3^ School of Life Sciences Shanghai University Shanghai China

**Keywords:** disorders of consciousness, FDG, glucose metabolism pattern, positron emission tomography, SSM/PCA

## Abstract

**Aims:**

The patient being minimally conscious state (MCS) may benefit from wake‐up interventions aimed at improving quality of life and have a higher probability of recovering higher level of consciousness compared to patients with the unresponsive wakefulness syndrome (UWS). However, differentiation of the MCS and UWS poses challenge in clinical practice. This study aimed to explore glucose metabolic pattern (GMP) obtained from ^18^F‐labeled‐fluorodeoxyglucose positron emission tomography (^18^F‐FDG‐PET) in distinguishing between UWS and MCS.

**Methods:**

Fifty‐seven patients with disorders of consciousness (21 cases of UWS and 36 cases of MCS) who had undergone repeated standardized Coma Recovery Scale‐Revised (CRS‐R) evaluations were enrolled in this prospective study. ^18^F‐FDG‐PET was carried out in all patients and healthy controls (HCs). Voxel‐based scaled subprofile model/principal component analysis (SSM/PCA) was used to generate GMPs. The expression score of whole‐brain GMP was obtained, and its diagnostic accuracy was compared with the standardized uptake value ratio (SUVR). The diagnostic efficiency was validated by one‐year later clinical outcomes.

**Results:**

UWS‐MCS GMP exhibited hypometabolism in the frontal–parietal cortex, along with hypermetabolism in the unilateral lentiform nucleus, putamen, and anterior cingulate gyrus. The UWS‐MCS‐GMP expression score was significantly higher in UWS compared to MCS patients (0.90 ± 0.85 vs. 0 ± 0.93, *p <* 0.001). UWS‐MCS‐GMP expression score achieved an area under the curve (AUC) of 0.77 to distinguish MCS from UWS, surpassing that of SUVR based on the frontoparietal cortex (AUC = 0.623). UWS‐MCS‐GMP expression score was significantly correlated with the CRS‐R score (*r* = −0.45, *p* = 0.004) and accurately predicted the one‐year outcome in 73.7% of patients.

**Conclusion:**

UWS and MCS exhibit specific glucose metabolism patterns, the UWS‐MCS‐GMP expression score significantly distinguishes MCS from UWS, making SSM/PCA a potential diagnostic methods in clinical practice for individual patients.

## Graphical abstract

Fifty‐seven chronic DoC patients and 30 HCs underwent ^18^F‐FDG PET/CT scans. GMPs were identified and quantified using SSM/PCA. UWS‐MCS‐GMP expression score achieved an area under the curve of 0.77 to distinguish MCS from UWS and significantly correlated with the CRS‐R score.

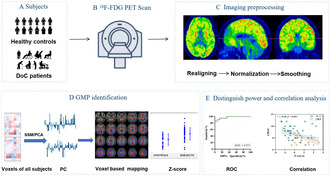

## 1 INTRODUCTION

Various causes including traumatic brain injury, intracerebral hemorrhage, and ischemic stroke can lead to disorders of consciousness (DoC). Chronic DoC, defined as a subacute‐to‐chronic period of >28 days after injury,^1^ manifests with alterations in arousal and/or awareness, prominently featuring unresponsive wakefulness syndrome (UWS) and minimal consciousness state (MCS). Although there is consensus advocating for more aggressive treatment for both UWS and MCS patients, studies indicate that only about 40% of patients may derive benefits from interventions aimed at arousal, with MCS patients being the primary target population.^2^, ^3^ Moreover, the rate of regaining effective verbal communication or functional object use is higher in MCS patients compared to those with UWS (48% vs. 14%).^4^, ^5^ However, the mechanisms of consciousness damage and recovery are still unclear, and the absence of a “gold standard” for detecting conscious awareness poses challenges in accurately identifying unequivocal signs of consciousness, impeding the formulation of individualized rehabilitation strategies.^2^

Despite the establishment of standardized behavioral assessments for MCS diagnosis, around 40% of UWS patients had evidence of residual consciousness by neuroimaging assessment.^6^, ^7^, ^8^ Structural quantitative analysis and functional connectivity of brain networks through multiparameter magnetic resonance imaging have gained understanding of consciousness processes. However, its low repeatability and its sensitivity to motion artifacts are major hurdles, and it has faced difficulties to translate mechanistic insights into clinically relevant information. Quantitative electroencephalography (EEG) shows high sensitivity in the diagnosis of residual consciousness, but it also has shortcomings such as poor specificity, low spatial resolution, and technical artifacts.^9^ Given that glucose metabolism accounts for over 95% of the primary energy source for neurons,^10^ ^18^F‐labeled‐fluorodeoxyglucose positron emitting tomography (^18^F‐FDG PET) imaging, which measures glucose metabolism, is a potential tool for detecting brain function related to residual consciousness. Early studies scaled regional glucose metabolism to a global mean and showed that preserved frontoparietal cortex metabolism with a sensitivity of 93% in identifying MCS and obtained an overall outcome prediction accuracy of 74%.^11^ However, the variable preservation of metabolism may be related to network activation associated with specific behavioral or perceptual functions in MCS patients.^12^ Stender et al.^13^ proposed that the level of consciousness correlates with whole‐brain energetic state and demonstrated the average cortical metabolic activity, in the best‐preserved hemisphere, with an accuracy of 84% to classify patients with chronic DoCs. However, maintenance of consciousness was not only dependent on the cortex, cortical–subcortical network is interconnected, and the metabolic activity and functional connectivity within DMN networks increase as patients transition from UWS to MCS. Lant et al.^14^ showed that the damage of the fibers' integrity connecting the anterior forebrain mesocircuit and the default mode network is more severe in UWS patients than those who are at least minimally conscious. Crone et al.^15^ demonstrated that the local efficiency of medial parietal differed between MCS and UWS, and alterations in the thalamus were particularly evident in non‐conscious patients. Therefore, showing cortical–subcortical interaction using ^18^F‐FDG PET may explain the interconnected functional networks crucial for the generation and maintenance of consciousness, thereby improving the diagnosis of DoC.

The scaled subprofile model/principal component analysis (SSM/PCA) technique is a multivariate statistical method that captures covariance patterns of voxel‐based differences in brain metabolism.^16^ This feature extraction method enhances the identification of significant patterns and mirrors the underlying relationships between brain regions. SSM/PCA has successfully identified specific cortical–subcortical interactions in Parkinson's syndromes^17^ and other neurodegenerative diseases,^18^ achieving superior classification through the derived glucose metabolism pattern (GMP). Thus, considering the network integrity of DoC patients, SSM/PCA can evaluate the changes at the network level, which is helpful for us to study the specific metabolic patterns of DoC and assist in diagnosis. We hypothesize that SSM/PCA can detect subtle variations between UWS and MCS, with the corresponding scores obtained used to diagnose residual consciousness. In the present study, we extracted GMPs from ^18^F‐FDG PET images and generated GMP expression scores for each participant as a potential index to characterize consciousness level. The objectives of this study were twofold: (1) perform SSM/PCA to generate GMPs in patients with DoC and (2) evaluate the potential clinical applications of GMPs to enhance diagnostic accuracy.

## 2 MATERIALS AND METHODS

### 2.1 Participants

Chronic DoC patients assessed with ^18^F‐FDG PET between January 2013 and December 2022 at Xijing Hospital were included. The consciousness level was determined through a comprehensive assessment, Revised Coma Recovery Scale (CRS‐R) was conducted over 5 consecutive days by a trained neuropsychologist, and the highest result was recorded.^4^ In case of ambiguity or disagreement between examiners, the preservation of frontoparietal cortex activity on ^18^F‐FDG PET was visually assessed by two nuclear medicine physicians. A diagnosis of UWS was made if there was complete bilateral hypometabolism in the associative frontoparietal cortex with preserved metabolism. Conversely, incomplete hypometabolism and partial preservation of activity within these areas led to a diagnosis of MCS.^11^ Discrepancies were resolved by a third physician, if necessary. In instances of any remaining ambiguity or disagreement within the team, patients underwent re‐assessment until a consensus was achieved.

Inclusion criteria were as follows: (1) The level of consciousness was accurately diagnosed through the comprehensive assessment mentioned above; (2) ≥28 days after brain injury event; (3) age >18 years. The exclusion criteria were as follows: (1) large focal brain damage involving more than two‐thirds of one hemisphere, severe deformation caused by surgery or trauma, as recorded by a certified neuroradiologist, (2) suboptimal image quality (motion of more than 3 mm in translation and 3° in rotation).

Thirty age‐ and gender‐matched healthy controls (HCs) (22 males, mean age ± standard deviation (SD) 48.19 ± 9.9 years) free of psychiatric or neurologic disorders on the basis of a health screening measure were also enrolled.

The prospective study was approved by the Ethics Committee of Xijing Hospital (No. KY20243056‐1), and written informed consent was obtained from the legal guardians of all participating patients and all HCs.

### 2.2 ^18^F‐FDG PET Scan

Interictal PET data were obtained using a PET/CT scanner (United Imaging, uMI780). The patients were required to fast for >6 h, and the level of fasting blood glucose was <11.1 mmol/L. ^18^F‐FDG with radiochemical purity of >95% was provided, and the injection dosage was calculated based on the patient's body weight (3.7 MBq/kg). All patients rested in a quiet environment for 40 min, with eyes open and head still. No patient used sedative or anesthetic drugs to avoid motion artifacts during the scanning. A low dose attenuation correction CT was conducted including the entire skull with the following scan parameters: tube voltage: 120 kV, and tube current: 200 mA with 6.2 mm axial resolution, enabling 47 contiguous transverse sections of the brain of 3.27 mm thickness. All images were reconstructed at 0.5‐mm slice thickness and 0.5‐mm increments. The attenuation‐corrected axial PET images were reconstructed with a 3D ordered‐subset expectation maximization (OSEM) algorithm (8 iterations and 32 subsets, 3‐mm cutoff).

### 2.3 Image preprocessing

Preprocessing was performed using statistical parametric mapping 12 software (SPM12, Department of Imaging Neuroscience, Institute of Neurology, London, UK) implemented in MATLAB R2018b (Mathworks Natick, MA, USA), including format conversion, realignment, spatial standardization, and smoothing. First, all DICOM images were converted into NIfTI format. Then, head movement correction of images was performed. Next, each PET image was co‐registered to the standard T1 image, and the co‐registered PET image was normalized to Montreal Neurological Institute (MNI) standard space with 2 × 2 × 2 mm^3^ voxel size. Finally, the images were smoothed with an 8 mm full width at half maximum Gaussian kernel to increase signal‐to‐noise ratios. A standard gray matter mask was applied to the preprocessed PET images to remove unrelated voxels.^19^

Regions of interest (ROI) were defined by the AAL template to calculate SUVR.^20^ ROI were divided into frontal and parietal, and a meta ROI was composed of the frontoparietal cortex. The whole brain served as the reference brain region.

### 2.4 SSM/PCA

**FIGURE 1:**
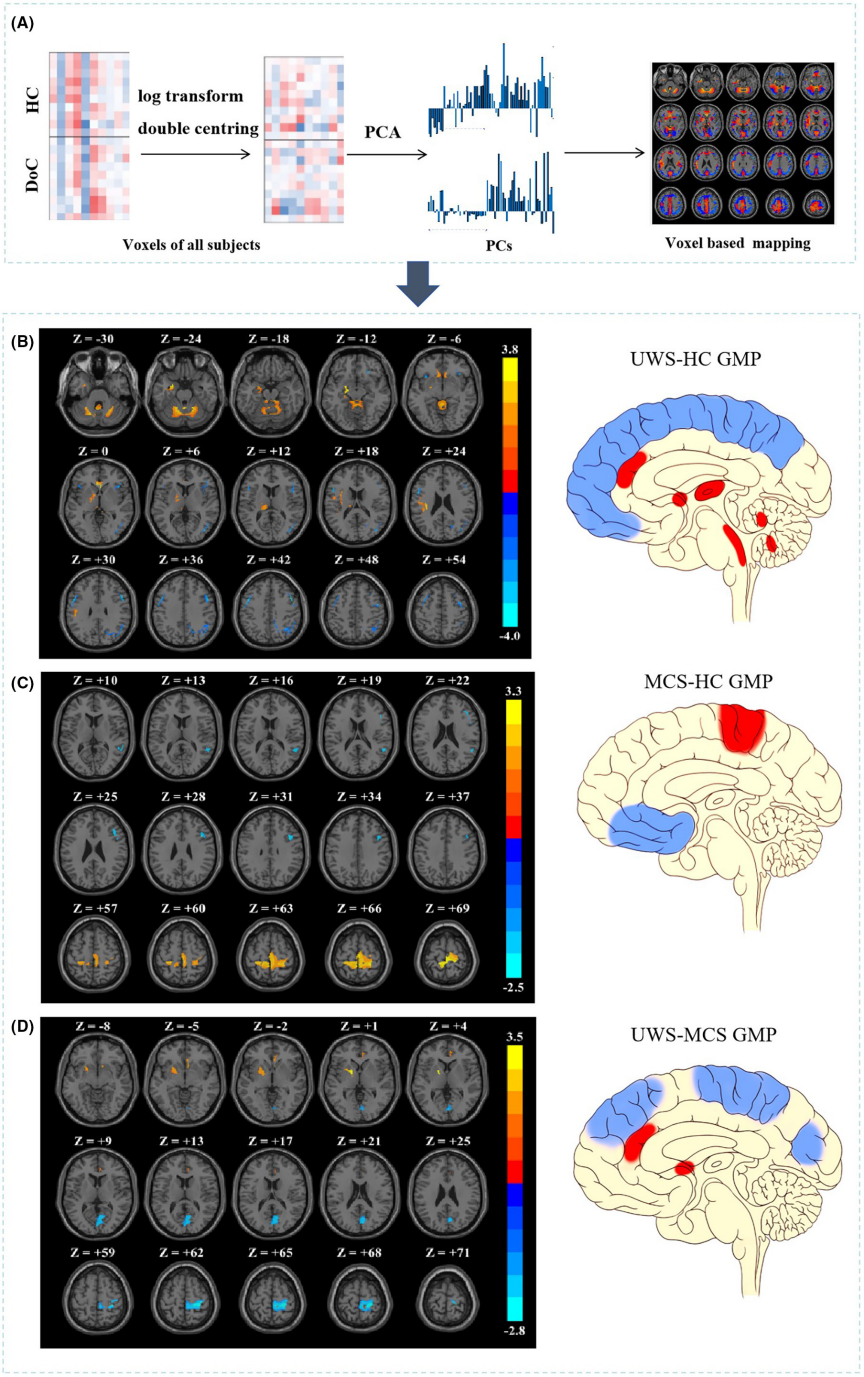
GMPs generated to disease group and reference. (A) GMPs generated using the SSM/PCA method. (B) UWS‐HC GMP, (C) MCS‐HC GMP, (D) UWS‐MCS GMP. Display of the SSM‐PCA results overlaid on a template in MNI space. Warm colors (yellow and red) indicate positive voxel weights (relative hypermetabolism), and the cool color (blue) indicates negative voxel weights (relative hypometabolism). The following is a schematic diagram of the corresponding glucose metabolic patterns.

Pattern analysis using scaled subprofile model and principal component analysis (SSM/PCA) was performed using the ScAnVP 7.0w package (http://www.feinsteinneuroscience.org) and implemented in MATLAB R2018b.^19^ A 35% threshold of the whole‐brain intensity maximum^18^ was applied to remove out‐of‐brain voxels, followed by a logarithmic transformation of the mean glucose metabolism within each voxel for every subject. Subsequently, principal component analysis (PCA) was invoked, characterizing the dataset by identifying independent and orthogonal principal components (PCs). The selection criteria for meaningful PCs were twofold: (1) the PCs explaining the top 50% variance; (2) the two‐sample *t*‐test of express score was *p* < 0.2 to ensure the inclusion of important PCs. The PCs that met all the above criteria were selected for logistic regression and generated comprehensive pattern and expression scores. In the comprehensive expression pattern, voxels with a threshold of |*z*| ≥ 1.65 (*p* ≤ 0.05) were selected, and the cluster level was set above 100.^18^, ^21^ Finally, the computation of subject expression scores was accomplished through utilization of a topographic profile rating algorithm. The detailed procedure is shown in Figure 1A.

The above processes were conducted on three groups: UWS and MCS were used as disease groups respectively, and HC was used as the control group to obtain the UWS‐HC‐GMP and MCS‐HC‐GMP. UWS was used as the disease group and MCS was used as the control group to obtain the UWS‐MCS‐GMP.

### 2.5 Statistical analysis

Demographic and neuropsychological data were summarized as numbers for categorical variables and analyzed by chi‐square tests. For continuous variables (age, CRS‐R scores, the GMP expression scores, and the SUVR) conform to the normal distribution, express in means ± SD, then analyzed by two‐sample t‐tests. For continuous variables (time from event occur to PET scan)not conform to the normal distribution, expression as median (quartile) and analyzed by non‐parametric tests.

To determine the sensitivity and specificity of the patterns, the AUC values of the ROC curves were used to evaluate the ability to distinguish power. The optimal cutoff point, balancing sensitivity and specificity, was determined by the Youden index method.

Pearson's correlation was used to explore the correlation between GMP expression scores and clinical CRS‐R. The rate of correctly predicted outcomes by GMP cutoff value was calculated. A *p* level of <0.05 was taken as statistically significant.

One year following the initial CRS‐R evaluation, Glasgow Outcome Scale‐Extended (GOS‐E) was conducted on patients to assess long‐term clinical outcomes. As we sought to predict the recovery of consciousness, the outcomes were categorized into “consciousness non‐recovery” (GOS‐E ≤ 2) or “consciousness recovery” (GOS‐E > 2).^11^, ^13^ Outcome evaluation was obtained from the patients' medical reports.

## 3 RESULTS

### 3.1 Subject characteristics

A total of 114 patients with disorders of consciousness caused by brain damage underwent ^18^F‐FDG PET/CT examinations at the Department of Nuclear Medicine, Xijing Hospital. Strict exclusion criteria were applied, leading to the inclusion of a refined sample comprising 57 patients (40 (70.2%) males, mean age ± SD 49.9 ± 17.1 years) in our study. Among these participants, 21 were diagnosed with UWS and 36 with MCS. Of these, 30 (52.6%) patients had a traumatic etiology, 21 (36.8%) had intracerebral hemorrhage or ischemic stroke, and 6 (10.5%) had toxic encephalopathy. The detailed clinical characteristics of DoC patients and HCs are shown in Table 1. No significant differences were observed between the chronic DoC patients and HCs for age or gender (*p* > 0.05). There were also no significant differences between the UWS and MCS groups for age (48.7 ± 16.9 years vs. 50.7 ± 17.5 years, *p* = 0.67), gender (17 males vs. 23 males, *p* = 0.55), or the time from event occurrence to the PET scan (37(34) days vs. 36.5(42), *p* > 0.05). The CRS‐R was significantly lower in the UWS group compared to the MCS group (3.95 ± 1.53 vs. 7.50 ± 2.70, *p* < 0.001).

**TABLE 1:** Detailed clinical characteristics in DoC patients and HCs.

	HC (*n* = 36)	UWS (*n* = 21)	MCS (*n* = 36)	*p*
Gender (*n*, %)				0.877
Male	22 (73.3%)	35 (67.3%)	23 (65.7%)	
Female	8 (26.7%)	17 (32.7%)	12 (34.3%)	
Age (years, mean ± SD)	48.19 + 9.85	48.7 ± 16.8	50.7 ± 17.5	0.808^a^
CRS‐R scores at PET scan		3.95 ± 1.53	7.50 ± 2.70	0.001
Time from event occur to PET scan (days, median (quartile))		37 (34)	36.5 (42)	0.803^b^
Etiology (*n*, %)				0.563
Traumatic		10 (47.6%)	20 (55.6%)	
Non‐traumatic		11 (52.4%)	16 (44.4%)	

^a^ Variable analyzed by ANOVA.

^b^ Variable does not conform to Gaussian distribution, analyzed by non‐parametric tests.

### 3.2 GMP definition and comparisons

Compared with HC, UWS GMP exhibited wide‐spread hypometabolism in the bilateral frontoparietal cortex including in the inferior frontal gyrus, middle frontal gyrus, and inferior parietal lobule, coupled with hypermetabolism in the subcortical regions including the midbrain, unilateral parahippocampal gyrus, lentiform nucleus, thalamus, anterior cingulate gyrus and bilateral cerebellar hemispheres (Figure 1B).

In comparison to HC, MCS GMP exhibited hypometabolism in the unilateral inferior frontal gyrus and superior temporal gyrus, with hypermetabolism in the bilateral precentral gyrus (Figure 1C). In addition, negative voxel weights were predominantly distributed in the right hemisphere.

Compared with MCS, UWS GMP was characterized by hypometabolism in the frontal–parietal cortical, including the precuneus, precentral gyrus, postcentral gyrus, paracentral lobule, frontal lobe, and parietal lobe. Hypermetabolism was observed in the lentiform nucleus, putamen, and anterior cingulate gyrus (Figure 1D).

### 3.3 Comparison between GMP expression scores and SUVR in specific cortical areas

**FIGURE 2:**
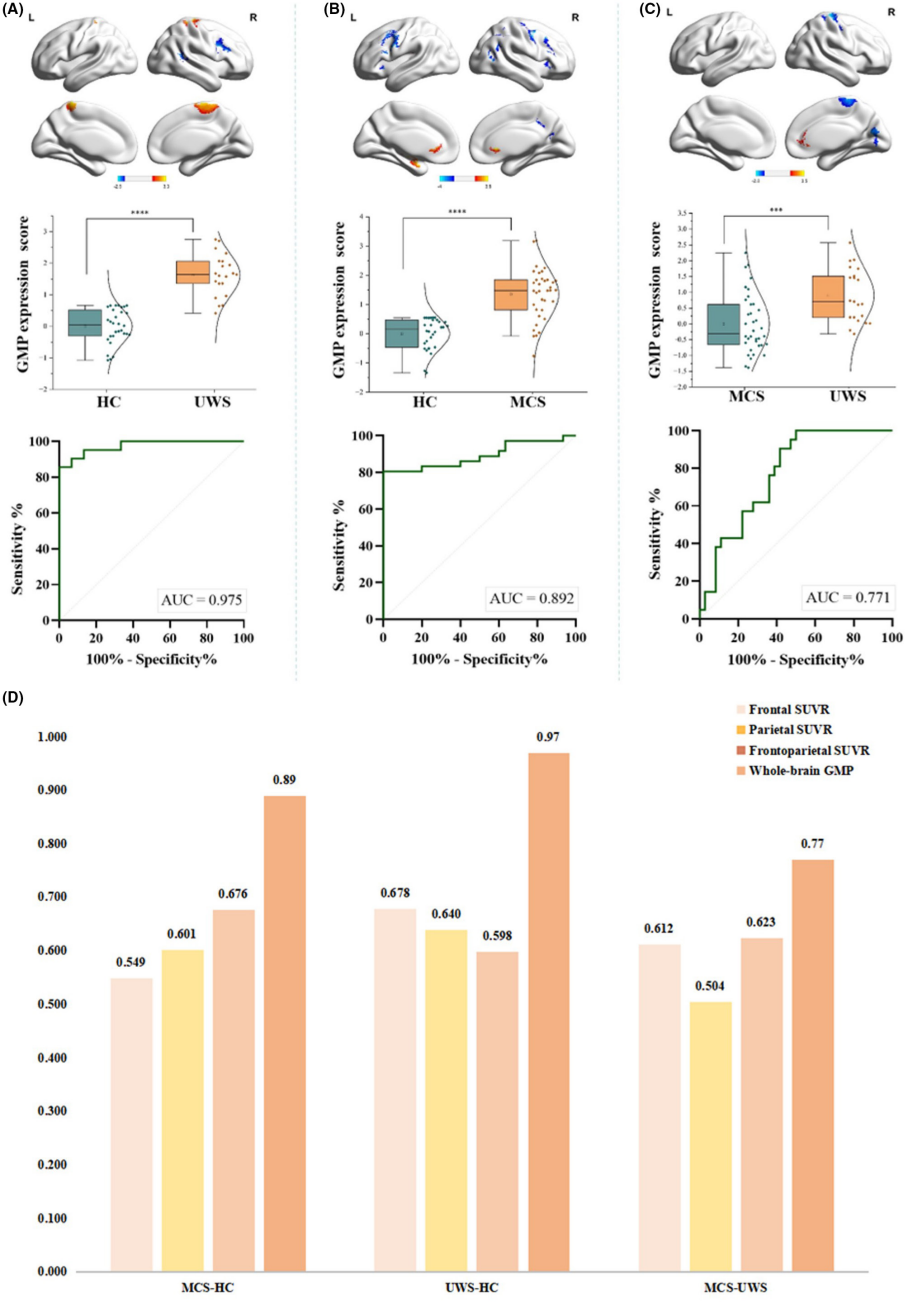
Comparison of GMP expression score and SUVR in diagnostic efficacy. (A) Distribution of subjects' scores for the UWS and HC groups, with the optimum discrimination threshold (empirical cut point = 0.781). ROC analysis of GMP scores compared between MCS and HC groups (AUC = 0.975). (B) Distribution of GMP scores of the MCS and HC groups, with the optimum discrimination threshold (empirical cut point = 0.575). ROC analysis: comparison of GMP scores between MCS and HC groups (AUC = 0.892). (C) Distribution of individual GMP scores of the MCS and UWS groups, with the optimum discrimination threshold (empirical cut point = 0.351). ROC analysis: comparison of GMP scores of the MCS and UWS groups (AUC = 0.771). (D) Inter‐group discrimination efficiency: corresponding GMP scores are superior to cortex SUVRs. The numbers shown in the figure represent the AUCs.

Mean GMP expression score was significantly higher in the UWS compared to the HC group (1.62 ± 0.66 vs.0 ± 0.53, *p* < 0.001). ROC analysis to distinguish UWS from HC yielded an AUC of 0.975, with a cut‐off value of 0.781 and a Youden index of 0.857 (Figure 2A).

Mean GMP expression score was significantly higher in the MCS group compared to the HC group (1.35 ± 0.88 vs. 0 ± 0.53, *p* < 0.001). ROC analysis for distinguishing MCS from HC resulted in an AUC of 0.892, with a cut‐off value of 0.58, and a Youden index of 0.806 (Figure 2B).

Mean GMP expression score was significantly higher in the UWS group compared to the MCS group (0.90 ± 0.85 vs. 0 ± 0.93, *p* < 0.001). The classification tasks comparing UWS and MCS exhibited an AUC of GMP score of 0.77, with a cut‐off value of 0.351 for distinguishing MCS from UWS (Figure 2C).

GMP expression score AUCs were better in distinguishing between UWS and HC, MCS and HC, UWS and MCS groups, compared to the cortex SUVR, which is shown in Figure 2D.

### 3.4 Correlation analysis

**FIGURE 3:**
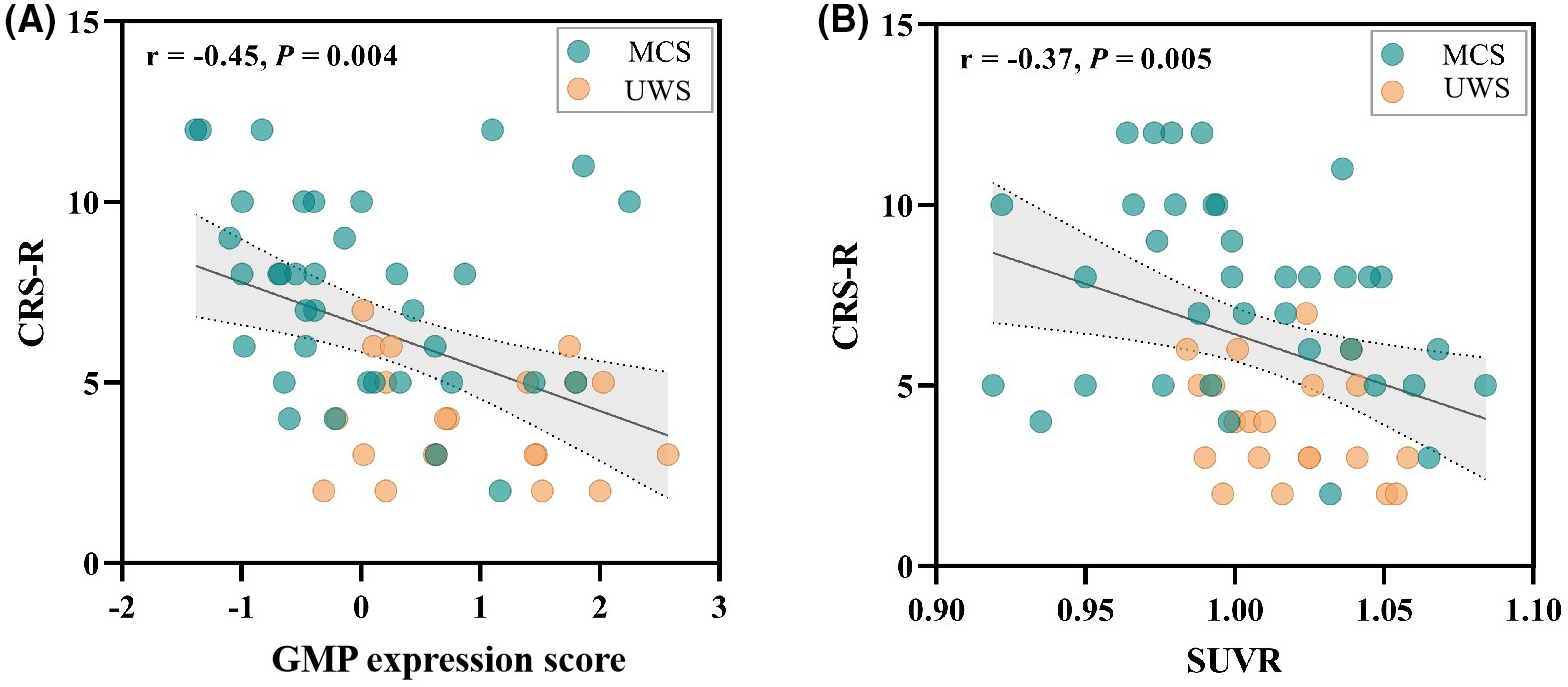
Pearson's correlations between GMP expression score, SUVR, and CRS‐R score. UWS‐MCS‐GMP expression score correlated with CRS‐R score (*r* = −0.45, *p* = 0.004). Frontoparietal cortex SUVR correlated with CRS‐R score (*r* = −0.37, *p* = 0.005).

The Pearson correlation indicated that UWS‐MCS‐GMP expression score correlated significantly with CRS‐R score (*r* = −0.45, *p* = 0.004) (Figure 3A).

There was no significant correlation between frontal lobe SUVR and UWS‐MCS‐GMP score (*r* = 0.05, *p* = 0.684), or between parietal lobe SUVR and UWS‐MCS‐GMP score (*r* = −0.12, *p* = 0.383).

However, frontoparietal cortex SUVR was significantly correlated with CRS‐R score (*r* = −0.37, *p* = 0.005) (Figure 3B).

### 3.5 Longitudinal follow‐up validation

**FIGURE 4:**
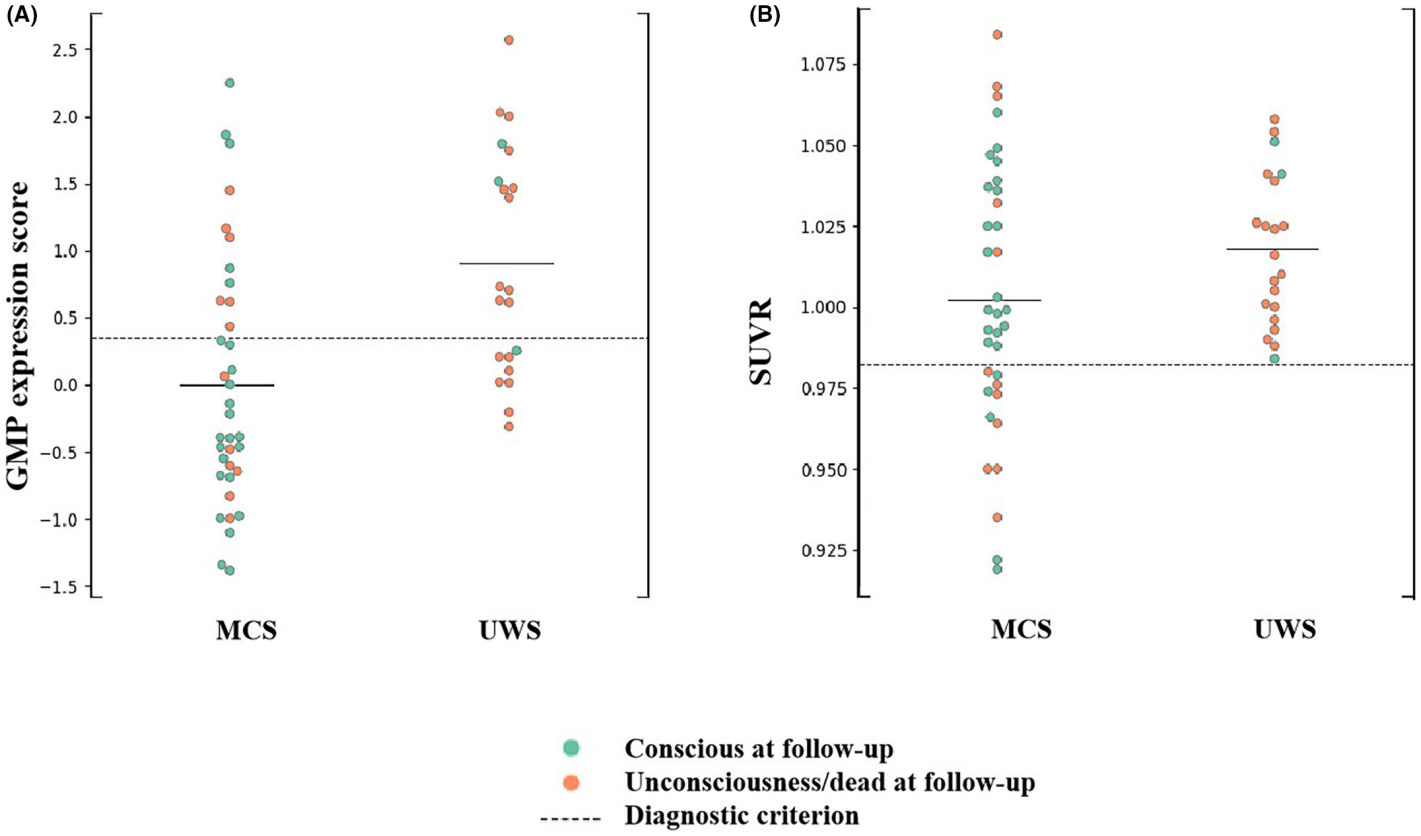
Longitudinal follow‐up validation by consciousness recovery. (A) Distribution of quantified GMP expression score for all individual patients in a pooled cohort. (B) Distribution of SUVR values for all individual subjects. The dashed line marks the optimal diagnostic cutoff between patients in UWS and MCS groups. Horizontal lines mark the group means.

Outcome data at 1‐year follow‐up were obtained for all the enrolled patients. Figure 4 shows group discrimination by the GMP expression score and SUVR based on frontoparietal cortex. GMP expression score correctly predicted 73.7% of outcomes, with 72.2% sensitivity and 76.2% specificity in manifesting consciousness at follow‐up (Figure 4A). Frontoparietal cortex SUVR correctly predicted 50.9% of outcomes, with the same sensitivity as GMP expression score, but only 14.3% specificity (Figure 4B).

A total of eight UWS patients had GMP scores below the 0.351 diagnostic threshold. Of these, three have recovered consciousness one year later. 13 UWS patients had GMP scores above the 0.351 diagnostic threshold: two with consciousness recovery. Of the other 11 MCS patients with GMP scores above the cut‐off value of 0.351, five patients maintained MCS or regained consciousness, whereas six patients presented with UWS at follow‐up.

## 4 DISCUSSION

The differentiation between MCS and UWS remains a substantial clinical challenge,^22^ necessitating the incorporation of neuroimaging biomarkers into the diagnostic process. In the present study, the role of ^18^F‐FDG PET with SSM/PCA was explored, revealing that MCS and UWS patients exhibit specific GMPs. Furthermore, the expression scores of these patterns could be used for individualized diagnosis of DoC in clinical practice.

SUVR‐based univariate analysis has demonstrated specific metabolic patterns, with MCS patients maintaining partial metabolism in the frontoparietal cortex, whereas UWS patients show a broad bilateral frontoparietal dysfunction.^11^ In contrast, the multivariate SSM/PCA employed in this study highlights voxels with positive or negative covariance, providing a more nuanced view of metabolism, from a pathophysiological standpoint. The MCS GMP, characterized by unilateral hypometabolism in the inferior frontal gyrus and superior temporal gyrus, aligns with univariate analysis.^11^ The bilateral hypermetabolism in the precentral gyrus may be indicative of purposeful motor behavior.

The UWS GMP included negative components in bilateral frontoparietal regions, consistent with previous univariate analysis.^11^ Positive components in the midbrain, lentiform nucleus, and thalamus highlighted the involvement of the ascending reticular activation system. The diagnosis of UWS is made when spontaneous eye‐opening re‐emerges, signaling a recovery of the reticular activating system.^1^ The significance of increased glucose metabolism in the limbic system, including the parahippocampal gyrus and the cingulate gyrus remains unknown but might signify compensatory/adaptive or maladaptive mechanisms, given the extensive connections of the limbic system with the cortex, thalamus, and brainstem.^23^ Inverse correlation in glucose metabolism between the cortical and subcortical regions including thalamus, lenticular nucleus, etc, in UWS patients may be indicative of a negative feedback mechanism in the “mesocircuit” model^24^ from an imaging perspective, suggesting that behavioral responsiveness, across different levels of DoC, may correlate with strong synergistic activation of the thalamus and frontoparietal network. The specific GMPs in UWS and MCS patients shown in present study suggested that disconnections in brain regions may impede the typical integration processes, and combined with the findings from previous basic experimental and functional MRI studies,^25^, ^26^ may indicate that level of consciousness is dependent on the integrity of widespread cortical–subcortical networks. Moreover, the different metabolic patterns associated with MCS and UWS underlying physiological or neurobiological mechanisms, which could help in refining diagnostic tools and developing targeted therapies.

Diagnosis accuracy in DoC is critical for designing appropriate care plans and establishing accurate prognoses. In our study, the UWS‐MCS‐GMP expression score, focusing on cortical–subcortical interactions, demonstrated significantly higher values in UWS than in MCS patients. Using a cut‐off value of 0.351, the study achieved an AUC of 0.77 in distinguishing MCS from UWS patients. This diagnostic efficiency surpassed that of SUVR calculations in the frontoparietal cortex (AUC 0.62) and slightly exceeded a previous study's AUC of 0.75 based on default mode network.^27^ While the discriminative power was lower than reported by Stender et al. (AUC of 0.82 and 0.84 based on overall cerebral metabolic rate^20^ and metabolic index of the best‐preserved hemisphere (MIBH),^13^ respectively), the differences could be attributed to variations in measurement methods, etiology, or uptake normalization approaches. The longitudinal follow‐up verification demonstrated that the GMP‐based criterion correctly predicted 73.7% of all patient outcomes. The reduced predictive efficacy might be attributed to high mortality, particularly in patients with MCS. A proportion of patients initially diagnosed with MCS converted to UWS during follow‐up, potentially linked to delayed interventions. Therefore, for patients diagnosed with MCS by GMP, active wake‐up therapy and long‐term care should be given, to reduce the inability to recover consciousness and even cognitive function caused by untimely treatment. Unfortunately, although GMP successfully identified 13 patients with no consciousness, three of them regained consciousness. A larger cohort with additional variables associated with the outcome is needed to validate the methods in the future.

We acknowledge several limitations of this study. Firstly, it had a relatively small sample size, moreover, the specific settings in which the data were collected might impact the generalizability of the conclusions. Secondly, given the lack of an external validation group in the entire cohort, we applied longitudinal follow‐up data to validate the results. However, the long‐term clinical outcomes may be affected by rehabilitation therapy, medicine treatment, level of care, and so on. The complications, such as pressure ulcers, lung infections, urinary tract infections, and deep vein thrombosis of the lower extremities, also affect the outcomes of patients and even to death. Thirdly, the study highlighted the limited value of unimodal imaging in evaluating the classification of consciousness disorders. We suggest that the specific GMPs identified in MCS and UWS patients should be validated in future multimodal MRI structural and functional connectivity studies.

Our findings suggest that although UWS and MCS are a continuum in consciousness disorders, they exhibit specific glucose metabolism patterns. The multivariate statistical method that captures covariance patterns of voxel‐based differences in brain metabolism, which is objective and computationally simple, provides easily interpretable results to distinguish MCS from UWS. Overall, ^18^F‐FDG PET thus constitutes a strong diagnostic and prognostic marker in DoC suffering from brain injury.

## AUTHOR CONTRIBUTIONS

Kun Guo: Drafting/revision of the manuscript for content, including medical writing for content; interpretation of data. Qi Zhang: Drafting/revision of the manuscript for content, including medical writing for content; Major role in the analysis of data. Zhiyong Quan: Major role in the interpretation of data. Yirong Wang: Organization and copying of data. Taoqi Ma: Acquisition of data. Jiehui Jiang: Major role in the analysis of data. Fei Kang: Major role in the interpretation of data. Jing Wang: Study concept or design.

## FUNDING INFORMATION

This study was supported by the National Natural Science Foundation of China (grant number. 92259304, 82122033, 82202208, 82171977) and Ministry of Science and Technology Foundation of China (grant number. 2022ZD0208000).

## CONFLICT OF INTEREST STATEMENT

The authors declare that they have no conflict of interest.

## Data Availability

The data that support the findings of this study are available from the corresponding author upon reasonable request.
